# In sea trout, the physiological response to salmon louse is stronger in female than in males

**DOI:** 10.1093/conphys/coac078

**Published:** 2023-01-12

**Authors:** Per Gunnar Fjelldal, Sussie Dalvin, Mathias Stølen Ugelvik, Audun Østby Pedersen, Tom J Hansen, Bjørnar Skjold, Lise Dyrhovden, Ann Kathrin Kroken, Ørjan Karlsen

**Affiliations:** Reproduction and Developmental Biology, Institute of Marine Research (IMR), Matre Research Station, 5984 Matredal, Norway; Pathogens and disease transfer, Institute of Marine Research (IMR), PO Box 1870, Nordnes, 5817 Bergen, Norway; Pathogens and disease transfer, Institute of Marine Research (IMR), PO Box 1870, Nordnes, 5817 Bergen, Norway; Reproduction and Developmental Biology, Institute of Marine Research (IMR), Matre Research Station, 5984 Matredal, Norway; Reproduction and Developmental Biology, Institute of Marine Research (IMR), Matre Research Station, 5984 Matredal, Norway; Pathogens and disease transfer, Institute of Marine Research (IMR), PO Box 1870, Nordnes, 5817 Bergen, Norway; Reproduction and Developmental Biology, Institute of Marine Research (IMR), Matre Research Station, 5984 Matredal, Norway; Reproduction and Developmental Biology, Institute of Marine Research (IMR), Matre Research Station, 5984 Matredal, Norway; Reproduction and Developmental Biology, Institute of Marine Research (IMR), PO Box 1870, Nordnes, 5817 Bergen, Norway

**Keywords:** sea trout, salmon louse, osmoregulation, hepatosomatic index, hatchery reared, cardiosomatic index, brown trout, Anadrome

## Abstract

The aims of this study were to compare male and female sea trout (*Salmo trutta*) with respect to their hypo-osmoregulatory ability over a simulated migration to seawater and their clinical and physiological response to salmon louse (*Lepeophtheirus salmonis*) infection in seawater and over a simulated pre-mature return to freshwater. For this purpose, 2-year-old hatchery-reared male and female brown trout (F1 offspring of wild caught anadromous fish) were infected with salmon lice and measured for changes in plasma ions, glucose, lactate and osmolality and relative heart, liver and gonad sizes during a simulated seawater migration and thereafter a premature return to freshwater after 4 weeks in seawater (pre-adult louse). Un-infected trout served as control. Male trout used longer time to develop full hypo-osmoregulatory ability in seawater and showed a stronger response in plasma glucose and lactate following simulated premature return to freshwater, compared to female trout. Response to salmon louse was stronger in female trout, shown by stronger osmotic stress by chalimus (plasma Cl^−^) and pre-adult louse (plasma osmolality) and elevated relative liver size (hepatosomatic index) by pre-adult louse in female compared to male trout. Moreover, high plasma cortisol in infected female and low plasma cortisol in infected male trout produced a significant treatment–sex interaction on plasma cortisol. Lice infection intensity was initially higher in male (0.18 lice g^−1^) compared to female trout (0.11 lice g^−1^) at the chalimus stage, but equal between sexes at the pre-adult stage (male 0.15 and female 0.17 lice g^−1^). This study showed that female trout were better adapted for changes in water salinity, while male trout were more robust against salmon louse infection. These results suggests that the elevated salmon louse infection pressure generated by salmon farming have strong and unexplored negative effects on wild sea trout populations. Further research on this topic is vital for the conservation of wild sea trout populations.

## Teaser

Female trout were better adapted for changes in water salinity, while male trout were better adapted for salmon louse infection. These findings may suggest a skewed sex ratio against more females among louse infected wild sea trout that returns pre-maturely to freshwater.

## Introduction

Brown trout is a native salmonid in Europe, North Africa and western Asia, but has also been intentionally introduced worldwide (MacCrimmon *et al.,* 1970). It pre-dominantly spawns in running freshwater where the female covers the fertilized eggs with stones and gravel (Ottaway *et al.,* 1981). Brown trout display large phenotypic plasticity that involves a wide range of different life history strategies, mainly as freshwater or anadromous fish (Jonsson, 1985, 1989; Caballero *et al.,* 2013). Anadromous brown trout—sea trout—migrates to seawater in the spring as first-time (smolts) or veteran (adults) migrants (Thorstad *et al.,* 2016) and heads back to freshwater the first autumn or after 2 or more years (Klemetsen *et al.,* 2003). Normal age for first-time migrants ranges between 1 and 8 years (Thorstad *et al.,* 2016).

Many wild sea trout populations are in a poor state caused by salmon louse. In a study of 403 sea trout populations in Norway, only 20% of the population were classified to be in a good or very good state, whereas 48% of the population were in a poor or very poor state (Thorstad *et al.,* 2019). Among negative anthropogenic impact factors on sea trout populations, salmon louse was thought to be by far the largest, followed by hydropower regulation, agriculture, road crossings, overexploitation and habitat alternation (Thorstad *et al.,* 2019). Salmon louse is a marine ectoparasitic crustacean copepod that attaches to its anadromous salmonid hosts, such as sea trout, as copepodid, and then go through several parasitic developmental stages before becoming a sexually mature adult louse: copepodid → chalimus I → chalimus II → pre-adult I → pre-adult II → adult (Hamre *et al.,* 2013). The mature adult female louse releases its progeny as nauplii, which then go through one more non-infective nauplii stage, before developing into infective copepodids. Lice larvae originating from adult female lice attached to farmed Atlantic salmon result in enhanced high infection pressure on wild sea trout (Thorstad *et al.,* 2015).

Salmon louse may affect host physiology, metabolism and organ sizes. On the host, the louse is sedentary until the pre-adult stage, when it changes morphology and starts to move around (Bjørn and Finstad, 1998; Hamre *et al.,* 2013). Feeding on sea trout by mobile pre-adult and adult lice may cause severe tissue damage and mortality for heavily infected fish (reviewed in Thorstad *et al.,* 2015). Salmon louse infection can also reduce relative liver size and increase relative heart size in Atlantic salmon (Medcalf *et al.,* 2021), but such effects have not been studied in sea trout. In addition to these clinical consequences of louse infection, physiological and metabolic consequences such as reduced hypo-osmoregulatory ability and liver glycogen content and elevated plasma cortisol, glucose and lactate have been reported in sea trout (Birkeland and Jakobsen, 1997; Wells *et al.,* 2006; Wells *et al.,* 2007). Minor osmoregulatory disturbance may arise already at the second chalimus stage in sea trout if infection intensity (II) is high, but severe osmoregulatory problems manifested as highly elevated plasma chloride and osmolality arise when the lice develop to the pre-adult and adult stages and may eventually cause death (Thorstad *et al.,* 2015).

To mitigate the problems caused by louse infection, sea trout perform premature returns to freshwater (Tully *et al.,* 1993; Birkeland, 1996; Birkeland and Jakobsen, 1997; Bjørn *et al.,* 2001; Serra-Llinares *et al.,* 2020). Salmon louse ultimately dies in freshwater (Finstad *et al.,* 1995; Wright *et al.,* 2016). While a laboratory study showed that simulated premature freshwater run of wild sea trout infected with salmon lice gave significant short-term physiological benefits across a range of osmoregulatory, metabolic and stress markers (Wells *et al.,* 2007), wild sea trout infected with salmon louse may also die after entering freshwater (Birkeland, 1996). The laboratory study by Wells *et al.* (2007) measured the physiology of the fish when the louse had reached the chalimus stage 5 days before return to freshwater, and then again 2 days after return to freshwater. Hence, the short-term response to freshwater in trout infected with more harmful mobile pre-adult salmon louse is unknown. Furthermore, possible effects of fish sex are unexplored. Skewed sex ratios with more females among anadromous and more males among resident fish has been reported in brown trout (Jonsson, 1985). Hence, it is plausible that male and female brown trout reacts differently to salmon lice, especially in laboratory studies where mixed sexed stocks with and equal sex ratio are subjected to seawater and infected with salmon louse. Knowledge on this topic is highly relevant for conservation practitioners wanting to understand the physiologically derived burden salmon louse can have on sea trout populations.

The main aims of this study were the following:

- Compare the louse II, and clinical (organ sizes) and physiological (plasma ions, cortisol, glucose, lactate) effects of salmon louse between female and male sea trout in seawater and over a simulated pre-mature return to freshwater.
- Investigate the hypo-osmoregulatory ability of male and female brown trout during a simulated seawater migration.

## Material and Methods

In this study, quadruple groups of newly seawater transferred mixed sex hatchery reared two-year-old brown trout (F1 offspring of wild sea trout) were either infected with salmon louse or kept as uninfected control. When the lice reached the pre-adult stage, water salinity was changed from salt water to freshwater to simulate a premature freshwater migration. The study lasted for 42 days and was terminated 7 days after the simulated premature river ascendance. Response parameters were mortality, growth, plasma sodium, chloride, osmolality, cortisol, glucose and lactate, as well as relative liver, gonad and heart size.

### Fish stock

Brown trout were offspring of wild fish caught as post-smolts near the outlet of the Matre River (western Norway). The parental fish were caught in fyke nets and transferred to indoor tanks, weaned onto pelleted salmon feed and reared under simulated natural photoperiod (Western Norway, 60^o^ N, 5^o^ E) in 9 C seawater until they reached sexual maturation. The mature fish were transferred to freshwater at natural temperature and simulated natural photoperiod and were stripped when the females ovulated. On 11 December 2019, eggs from 14 females were mixed and thereafter fertilized with milt from three males. The fertilized eggs were incubated on 8 C and first feeding was on 27 March 2020. The fish were reared at 12 C and continuous light until summer solstice 2020 when water temperature was changed to natural. Continuous light was replaced with simulated natural photoperiod on 1 October 2020.

### Ethical statement

All experiments were performed at the Institute of Marine Research (IMR), Matre Research Station (60° N, 5° E, Western Norway), which is authorized for animal experimentation (Norwegian Food Safety Authority, facility 110) in accordance with International guidelines, certified using Norwegian research permit number 21287.

### Experimental design

On 26 April 2021, the fish were randomly allocated to eight square white covered fiberglass tanks (1 × 1 × 0.43 m) with 45 fish in each, at natural temperature, freshwater and simulated natural photoperiod. On 3 May, all fish were sedated (0.1 g L^−1^ tricaine methanesulfonate; Finquel MS-222) and measured for fork length and body weight. Water salinity was gradually increased from 0.8 to 34 ppt during the period 3–7 May (Jonsson *et al.,* 1994). In parallel to the experimental activity, the outward migration of wild smolts from the Matre River was monitored daily; wild smolts were observed at the river outlet in the period 26 April to 10 May. The experimental fish and wild fish were from the same strain and experienced similar temperature and photoperiod in freshwater, and the time of change in water salinity for the experimental fish was based on the migratory behavior of the wild fish. On 10 May, when the fish had been at 34 ppt salinity for 3 days, four tanks were infected with salmon lice copepodids, while four tanks were kept as uninfected controls. In all eight tanks (four infected, four uninfected) the water level was reduced to 10 cm depth, and water flow was stopped before adding copepodids (10 days post-hatch) to the four infection tanks. Then, in all tanks, normal water flow was resumed after 20 min. In total, 12 480 copepodids were used to infect the fish (3120 copepodids per tank), giving an overall average infection pressure of 83 lice per fish (number of fish per tank at infection ranged between 36 and 39). Salmon lice copepodids that were used for the infection challenge were produced according to standard methods (Hamre *et al.,* 2009) at ~8C, sourced from IMR’s in-house production stock. Lice were transferred to the experimental facilities in cooler boxes, and infection challenges occurred on the same day as lice were transferred. The seawater temperature was 9 C, and photoperiod simulated natural throughout. On 7 June, the lice had reached the pre-adult stage and water salinity was decreased back to 0.8 ppt, and the experiment was terminated on 14 June 2021.

**Table 1:** Number (*n*) of female and male trout sampled at the different sampling points (S1–S6)

Sampling	Female trout (*n*)		Male trout (*n*)	
	Control	Infected	Control	Infected
S1^a^	15		9	
S2^a^	12		12	
S3	4	6	8	6
S4	17	22	23	18
S5	23	14	17	26
S6	26	27	14	13

^a^Before infection with salmon louse.

**Figure 1:**
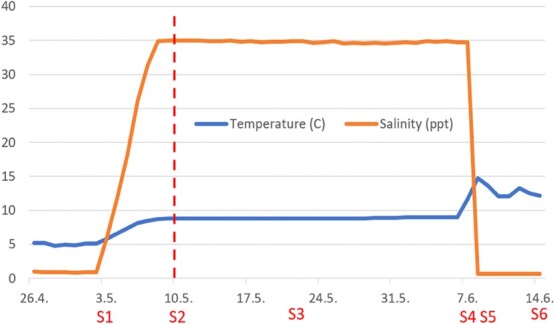
Temperature and salinity profiles during the experimental period. Sampling points are indicated by S1–S6. Vertical dotted line indicates time of louse infection. S1 = 03 May (Day 0), freshwater sampling before gradual increase in salinity from 0.8 to 34 ppt. S2 = 10 May (Day 7), time of lice infection, after 72 h at 34 ppt. S3 = 21 May (Day 18), chalimus stage louse. S4 = 07 June (Day 35) pre-adult stage louse seawater sampling, before change from 34 to 0.8 ppt. S4 = 09 June (Day 37), 48 h after change from 34 to 0.8 ppt. S6 = 14 June (Day 42), 7 days after change from 34 to 0.8 ppt.

### Samplings

Fish were sampled on 3 May (in freshwater) (**S1**), 10 May (after 72 h at 34 ppt, before lice infection) (**S2**), 21 May (**S3**), 07 June (**S4**), 09 June (after 48 h at 0.8 ppt) (**S5**) and 14 June (**S6**). A total of 24 fish (3 per tank) were sampled on S1, S2 and S3, while 80 fish were sampled on S4 (10 per tank), S5 (10 per tank) and S6 (8–12 per tank). Sampling involved netting one fish at a time from their respective tanks, applying sedation (0.01 g L^−1^ metomidate hydrochloride; Aquacalm vet., Scan Aqua AS, Årnes, Norway) measuring fork length and body weight, sampling blood and dissection for sex determination and measurement of gonad, liver, heart and viscera weights. The number of female and male trout sampled at different sampling points are shown in Table 1.

Lice were also counted on S3–6. Counts of lice per fish included all lice remaining in individual anesthetic water containers that they were placed in. Louse development followed a normal speed according to temperature (Hamre *et al.,* 2019), and had reached the chalimus stage at S3 and pre-adult stage at S4. After counting lice, but before further sampling, the fish were euthanized following sedation by a sharp blow to the head. Blood was centrifuged and plasma stored at −80 C until analysed. Blood sample was taken from the caudal vessel with a heparinized tuberculin syringe fitted with a 25-gauge needle. An outline of the experimental design and sampling points are shown in Fig. 1, and II and success per rearing tank at the pre-adult stage (S4) are shown in Table 2.

### Plasma analysis

Plasma ion levels (Na^+^, Cl^−^) were detected with an ABL90 FLEX PLUS blood gas analyzer (Radiometer Medical ApS, Åkandevej 21, DK-2700, Brønshøj, Denmark). Plasma osmolality was determined by freeze point determination (Fiske microosmometer Model 210, Norwood, MA, USA). Plasma cortisol concentration was quantified with an ELISA assay kit (Demeditec Diagnostics GmbH, article number DEH3388) and a Sunrise microplate reader (Tecan).

### Calculations and statistical analysis

II was calculated as follows: II = Ln ÷ Fw, where Ln was number of lice on infected fish and Fw was body weight (g) of infected fish at time of counting lice.

The condition factor (CF) was calculated as follows: CF = (Fw × 100) ÷ Fl^3^, where Fw was body weight (g) and Fl was fork length (cm).

**Table 2:** Lice infection level at time of infection (S2) and lice II, abundance and success/survival (mean ± SE) on S4 (pre-adult); four tanks served as uninfected controls

Tank no.	Infection (copepodids fish^−1^)	II (lice g^−1^ fish)	Lice abundance (lice fish^−1^)	Infection success and survival (%)
1	87	0.15 ± 0.03	19 ± 4	22
2	82	0.18 ± 0.04	20 ± 4	24
3	82	0.14 ± 0.01	17 ± 3	21
4	80	0.16 ± 0.02	24 ± 3	30

Gonadosomatic index (GSI) was calculated as follows: GSI = (Gw *×* 100) ÷ Fw, where Gw was gonad weight and Fw body weight.

Hepatosomatic index (HSI) was calculated as follows: HSI = (Hw × 100) ÷ Fw, where Hw was liver weight and Fw body weight.

Cardiosomatic index (CSI) was calculated as follows: CSI = (Cw × 100) ÷ Fw, where Cw was heart weight and Fw body weight.

The data were analyzed using Statistica version 12 (StatSoft, Inc., 2300 East 14th Street, Tulsa, OK, USA). Results are shown as means with their standard errors.

**Figure 2:**
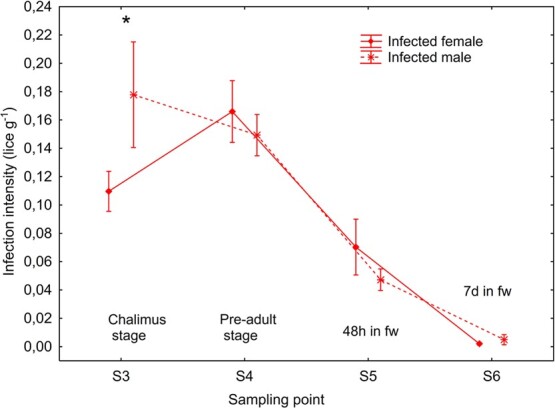
II (mean ± SE) in female and male brown trout infected with salmon louse. S3 is 11 days after infection when the louse had reached the chalimus stage. S4 is 28 days post-infection when the louse had reached the pre-adult stage, at which time point the water salinity was changed from salt water to freshwater to simulate pre-mature return to freshwater. S5 and S6 are after 2 and 7 days in freshwater, respectively. ^*^Significant difference within sampling points.

**Figure 3:**
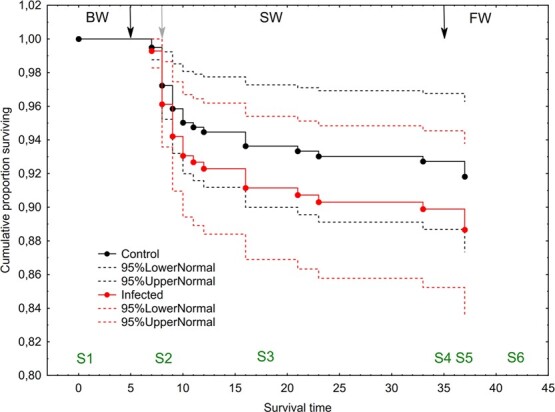
Survival plot for un-infected control and salmon louse infected brown trout. Water salinity was gradually increased from 0.8 to 34 ppt between 3 (S1) and 7 May, lice infection was on 10 May (S2), lice reached the pre-adult stage on 07 June (S4) when water salinity was changed from 34 to 0.8 ppt to simulate premature return to freshwater. Black arrows indicate when the fish went in and out of full salinity seawater (34 ppt). Gray arrow indicates time of lice infection. BW, brackish water (gradual increase from 0.8 to 34 ppt); SW, sea water; FW, freshwater.

Statistical analysis of mortality was done by Cox Proportional Hazards regressions using the counting process model.

To test if the four experimental tanks containing those fish to become controls and the four experimental tanks containing those fish to become infected with salmon lice had fish of equal size after distribution into the experimental tanks, possible significant differences in length, weight and CF were tested with a mixed model factorial ANOVA design with tank as random factor and group (to become control/infected) as fixed factor on S1 (3 May). Data from sampling S1 and S2 (test 1) were analysed together to study possible effects of fish sex and time (and their interactions) before lice infection and over the period with increasing water salinity. Data from samplings S2 to S6 (test 2) were tested together to study possible effects of fish sex, time, louse infection and their interactions. Significant differences in the response parameters length, weight, CF, HSI, CSI, GSI, plasma Cl^−^, Na^+^, glucose, lactate, osmolality and cortisol were tested using a mixed model factorial ANOVA design with tank as random factor (test 1 and 2) and sampling point (test 1 and 2), infection group (only test 2) and sex (test 1 and 2) as fixed factors. Possible differences in lice II over time and between sexes (within infected fish only) were tested using a mixed model factorial ANOVA design with tank as random factor and sampling point (S3–S6) and fish sex as categorial predictors. Significant ANOVAs were followed by Student–Keuls *post hoc* tests to defect possible differences between samplings, treatment groups and sexes.

**Figure 4:**
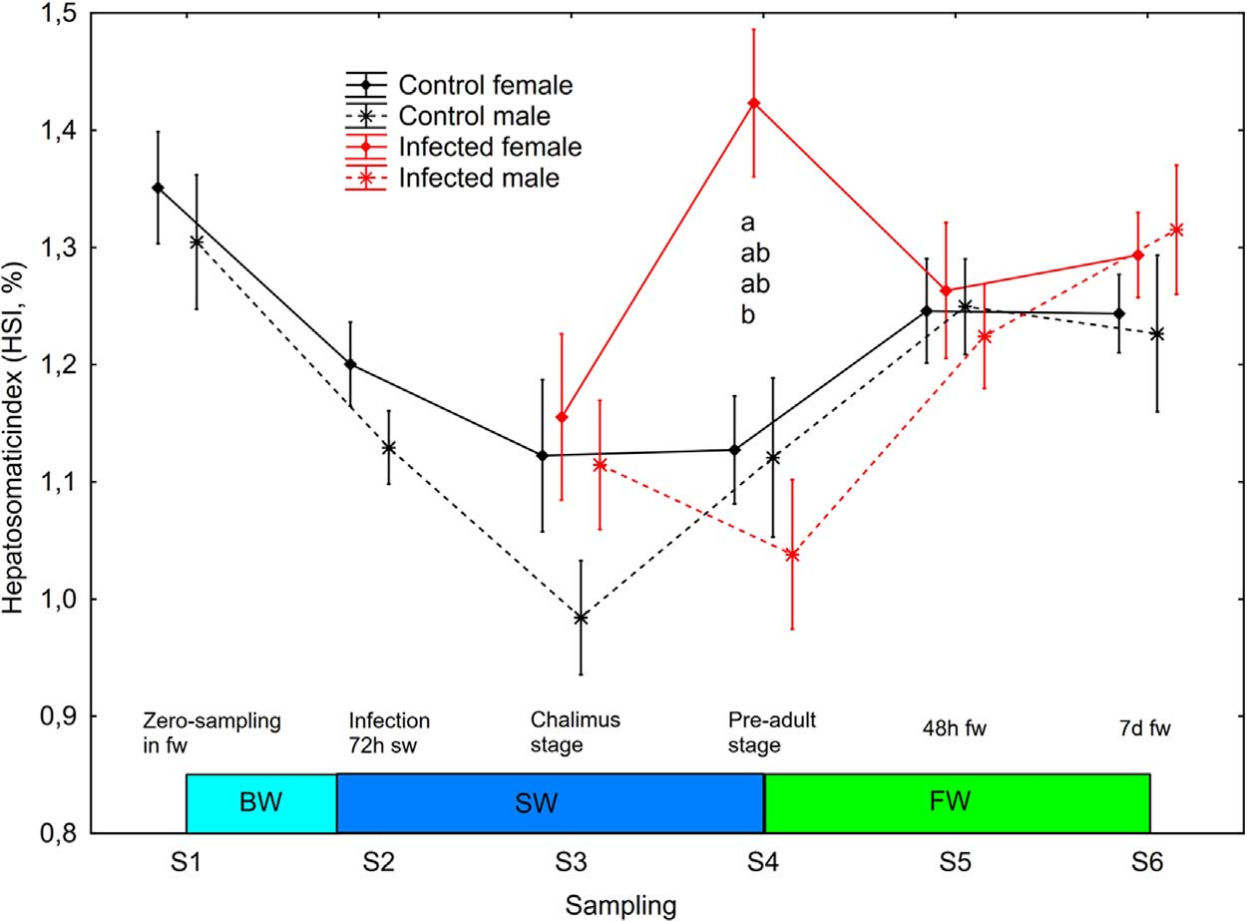
HSI (mean ± SE) in un-infected control and salmon louse infected female and male brown trout. Different lower-case letters denotes significant difference within sampling points. BW, brackish water (gradual increase from 0.8 to 34 ppt); SW, sea water; FW, freshwater.

## Results

### Higher II in male trout at the chalimus stage

There was a significant interaction between sampling point and sex (mixed model ANOVA, *P* = 0.0026) on lice II. Infected males had significantly higher (Newman–Keuls test, *P* < 0.05) II than females at S3 (chalimus stage) (Fig. 2). There was a strong trend (Newman–Keuls test, *P* = 0.0505) towards higher II at S4 (pre-adult) compared to S3 in females, both sexes had significantly lower II at S5 (48 h in fw) than S4 and lastly females but not males had significantly lower (Newman–Keuls test, *P* < 0.05) II at S6 compared to S5.

### No effect of lice on fish mortality

There was no difference in cumulative mortality between the control and infected groups over the experimental period (Cox Proportional Hazards regression, *P* = 0.066). Both groups showed a sharp increase in cumulative mortality over a 6-day period, starting after the fish had been in full strength seawater for 48 h (Fig. 3). Cumulative mortality was 3.3% after 72 h at 34 ppt seawater. Final cumulative mortality was 8.4% in the control group and 11.9% in the infected group.

### No effect of lice on fish growth

At the start of the experiment (3 May), all fish were measured for length and weight, and there were no differences (mixed model ANOVA) in length (*P* = 0.70), weight (*P* = 0.95) or CF (*P* = 0.12) between the four tanks containing the fish that formed the control group and the 4 tanks containing the fish that formed the infected group. Before lice infection, and over the period including gradual change from fresh- to seawater (S1–S2), there was a significant effect (mixed model ANOVA, *P* = 0.0035) of sampling point on CF where males had significantly higher (Newman–Keuls test, *P* < 0.05) CF than females at S1, and also a significant reduction in CF from S1 to S2 (Supplementary file 1). From the point of lice infection (S2) onwards, there was a significant effect (mixed model ANOVA, *P* = 0.0026) of sampling point on CF (Fig. 4), but not length (Supplementary file 2) and weight (Supplementary file 3).

### Males had bigger hearts while females developed larger livers in response to salmon lice infection

**Figure 5:**
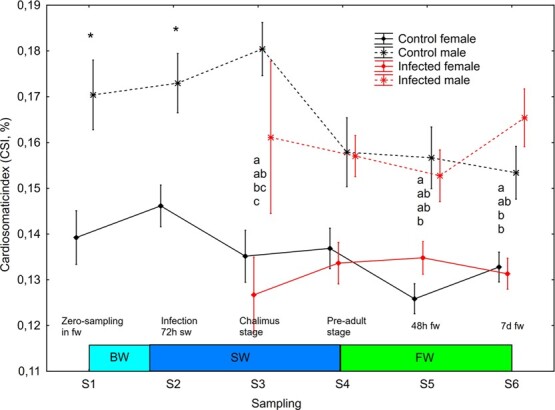
CSI (mean ± SE) in un-infected control and salmon louse infected female and male brown trout. ‘*’ and different lower-case letters denotes significant difference within sampling points between sexes (before louse infection, S1–2) and sex/treatment (after louse infection, S2-S6), respectively. BW, brackish water (gradual increase from 0.8 to 34 ppt); SW, sea water; FW, freshwater.

Before lice infection, and over the period including gradual change from freshwater to seawater (S1-S2), there was a significant effect (mixed model ANOVA, *P* = 0.011) of sampling point on HSI, and both males and females had significantly lower (Newman–Keuls test, *P* < 0.05) HSI on S2 than S1 (Fig. 4). From the point of lice infection (S2) onwards, the following significant effect (mixed model ANOVA) were observed: (i) treatment (*P* = 0.0075), sex (*P* = 0.0053) and sampling*sex (*P* = 0.0022) and sampling*sex*treatment (*P* = 0.0034) interactions on HSI (Fig. 4); (ii) sampling (*P* = 0.0080) and sex (*P* = 0.0005) on CSI (Fig. 5); (iii) sampling (*P* = 0.0335) and sex (*P* = 0.0187) on GSI (Supplementary file 4). HSI was significantly higher (Newman–Keuls test, *P* < 0.05) in infected females than infected males on sampling S4 (Fig. 4). CSI was significantly higher (Newman–Keuls test, *P* < 0.05) in control males than control and infected females, and in infected males than infected females at S3, in control males than control females at S5 and in infected males than control and infected females at S6 (Fig. 5).

### Males had slow acclimation of hypo-osmoregulatory ability while infected females struggled with plasma ion regulation

Seawater entry elevated plasma ions and lactate. Before lice infection, and over the period including gradual change from fresh- to seawater (S1-S2), there was a significant effect (mixed model ANOVA) of sampling point on plasma Na^+^ (*P* < 0.0001), Cl^−^ (*P* = 0.0001), osmolality (*P* = 0.0001) and lactate (*P* = 0.0048), which all increased significantly (Newman–Keuls *post hoc* test, *P* < 0.05) from S1 to S2 in both male and female trout.

From the point of lice infection in seawater (S2) until the final sampling in freshwater (S6) the following changes were observed in plasma Na^+^, Cl^−^, osmolality, cortisol, glucose and lactate.

#### Control males had higher plasma Na ^+^ than control females in seawater

**Figure 6:**
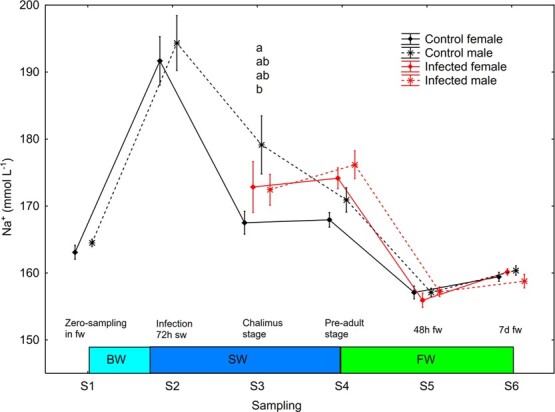
Plasma Na^+^ (mean ± SE) in un-infected control and salmon louse infected female and male brown trout. Different lower-case letters denote significant difference within sampling points. BW, brackish water (gradual increase from 0.8 to 34 ppt); SW, sea water; FW, freshwater.

There was a significant effect (S2–S6, mixed model ANOVA, *P* < 0.0001) of sampling point on plasma Na^+^. Plasma Na^+^ decreased significantly (Newman–Keuls *post hoc* test, *P* < 0.05) in all four sex/treatment groups between S2 and S3, was significantly higher in control male than female at S3, decreased significantly in control males between S3 and S4 and finally decreased significantly in all four sex/treatment groups after change from salt water to freshwater between S4 and S5 (Fig. 6).

#### Control males had higher plasma Cl ^−^ than control females in seawater and chalimus lice elevated plasma Cl^−^ in female but not male trout

**Figure 7:**
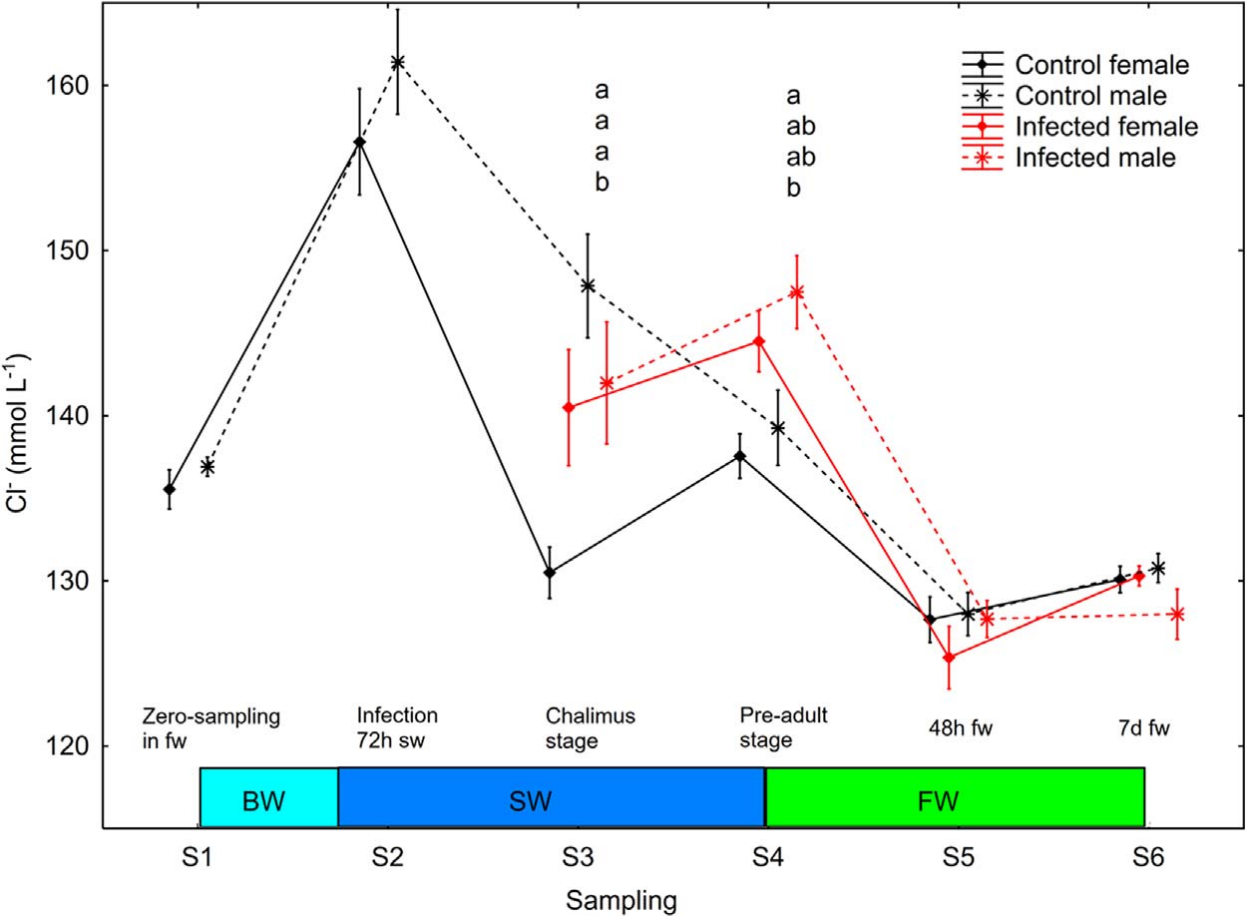
Plasma Cl^−^ (mean ± SE) in un-infected control and salmon louse infected female and male brown trout. Different lower-case letters denote significant difference within sampling points. BW, brackish water (gradual increase from 0.8 to 34 ppt); SW, sea water; FW, freshwater.

There was a significant effect (S2–S6, Smixed model ANOVA, *P* < 0.0001) of sampling point on plasma Cl^−^. Plasma Cl^−^ decreased significantly (Newman–Keuls *post hoc* test, *P* < 0.05) in all four sex/treatment groups between S2 and S3, was significantly lower in control females than control males and infected females at S3 (chalimus lice), increased significantly in control females from S3 to S4, and finally decreased significantly for all four sex/treatment groups at change from salt water to freshwater between S4 and S5 (Fig. 7).

#### Pre-adult lice elevated plasma osmolality in female but not male trout

**Figure 8:**
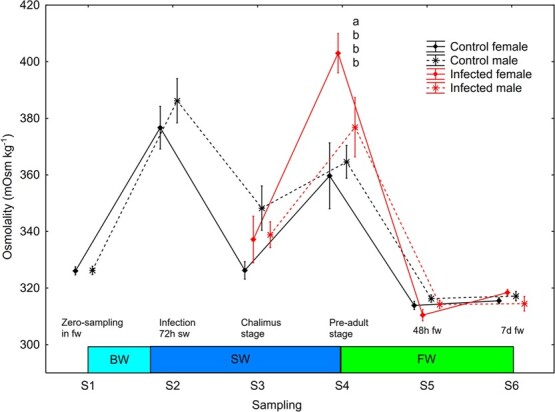
Plasma osmolality (mean ± SE) in un-infected control and salmon louse infected female and male brown trout. Different lower-case letters denote significant difference within sampling points. BW, brackish water (gradual increase from 0.8 to 34 ppt); SW, sea water; FW, freshwater.

There was a significant effect (S2–S6, mixed model ANOVA) of sampling point (*P* < 0.0001), treatment (*P* = 0.022) and a sampling point^*^treatment interaction (*P* = 0.0285) on plasma osmolality. Plasma osmolality decreased significantly (Newman–Keuls *post hoc* test, *P* < 0.05) in all four sex/treatment groups between S2 and S3, increased significantly between S3 and S4 in infected males and females and control females, was significantly higher in infected compared to control females at S4 (pre-adult lice), and finally decreased significantly in all four sex/treatment groups between S4 and S5 at change from salt water to freshwater (Fig. 8).

#### Lice had a stronger effect on plasma cortisol in female compared to male trout

**Figure 9:**
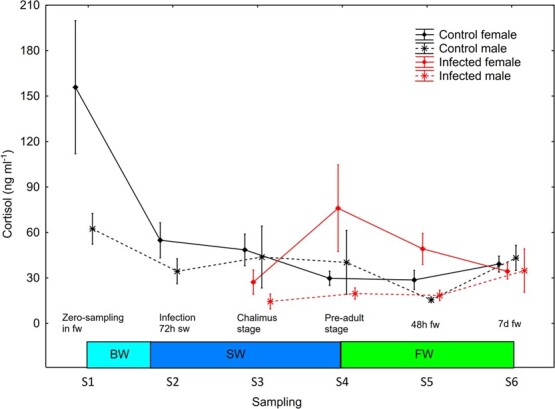
Plasma cortisol (mean ± SE) in un-infected control and salmon louse infected female and male brown trout. BW, brackish water (gradual increase from 0.8 to 34 ppt); SW, sea water; FW, freshwater.

Consequently, there was a significant (mixed model ANOVA) treatment*sex interaction (*P* = 0.0212) on plasma cortisol in the period S2–S6. There were, however, no significant differences (Newman–Keuls *post hoc* test, *P* > 0.05) between groups or sexes within sampling points or significant changes over time within groups in plasma cortisol (Fig. 9).

#### Plasma glucose and lactate increased in male trout at return to freshwater

**Figure 10:**
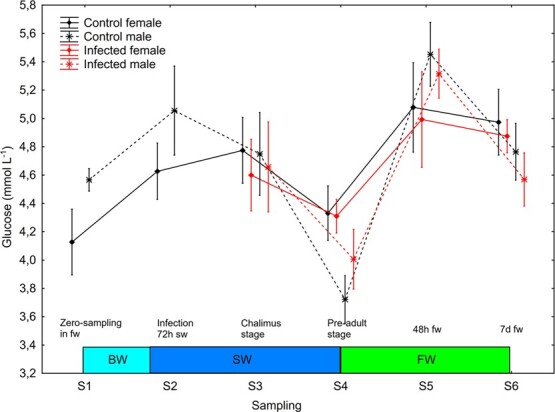
Plasma glucose (mean ± SE) in un-infected control and salmon louse infected female and male brown trout. BW, brackish water (gradual increase from 0.8 to 34 ppt); SW, sea water; FW, freshwater.

**Figure 11:**
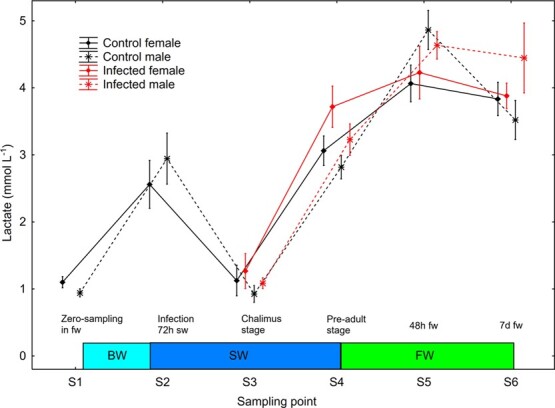
Plasma lactate (mean ± SE) in un-infected control and salmon louse infected female and male brown trout. BW, brackish water (gradual increase from 0.8 to 34 ppt); SW, sea water; FW, freshwater.

There was a significant effect (S2–S6, mixed model ANOVA) of sampling point on plasma glucose (*P* = 0.0002) and lactate (*P* < 0.0001). Plasma glucose increased significantly (Newman–Keuls *post hoc* test, *P* < 0.05) in control and infected males at change from salt water to freshwater between S4 and S5 (Fig. 10). Plasma lactate decreased significantly between S2 and S3 and increased significantly between S3 and S4 in all four sex/treatment groups, and between S4 and S5—change from salt to freshwater—in control and infected males (Fig. 11).

## Discussion

The present study is the first to show an effect of fish sex on the response to salmon louse. The observed increased HSI and plasma osmolality in female trout infected with pre-adult lice suggests that the lice caused stronger osmoregulatory and metabolic consequences in female compared to male trout.

### Male trout had higher chalimus lice II than female trout

The currently observed initial higher louse II in male than female trout at the chalimus stage may be related to an initial higher louse II or higher louse survival on male trout. The following increased II between the chalimus and pre-adult stages in female trout and equal II in male and female trout at the latter stage can be explained by movement of pre-adult louse between individual fish (Birkeland and Jakobsen, 1997).

A possible initial higher louse survival on male compared to female trout may be linked to that male trout used longer time to develop hypo-osmoregulatory ability in seawater. Such an effect may have reduced their ability to rid themselves of lice. Thereafter, when males were able to fine-tune their osmoregulation in concert with the lice developing to the mobile pre-adult stage, they may have started puberty as suggested by their higher CSI (Järvi *et al.,* 1991). Indeed, male brown trout mature at an earlier age than female (e.g. Nakari, 1997), and in the currently studied trout population, hatchery reared fish typically mature as 2-year-old males and 3-year-old females (authors own observations). Krasnov *et al.* (2015) found that both sexual maturation and administration of sex hormones (17β-estradiol and testosterone) in immature fish reduced lice infection in Atlantic salmon. Thus, onset of puberty in male trout may have induced a movement of pre-adult lice from male to female trout.

The current study did, however, not follow lice levels on the same individual fish over time, but sampled random fish that were euthanized. Further studies should examine lice levels over time in hatchery reared male and female trout in more detail, along with osmoregulation and plasma sex steroid levels. Such studies should include 3-year-old trout to explore possible effects of maturation in both sexes.

### The presence of chalimus lice elevated plasma chloride in female trout

Earlier studies have shown that sea trout may show minor osmoregulatory disturbance at high lice intensities when the louse moult to the second chalimus stage (Thorstad *et al.,* 2015). In this study, mean II at the chalimus stage was low and at 0.11 lice g^−1^ in female trout, but still infected females had higher plasma chloride levels than un-infected at this time point (S3). Uninfected females had low plasma chloride levels at the chalimus stage (S3). Thus, their hypo-osmoregulatory ability may have been ‘overstimulated’. Plasma chloride had increased and normalized two weeks later un-infected females (S4). Hence, the higher plasma chloride in infected than un-infected females at the chalimus stage was either caused by a reduced magnitude in the achievement of hypo-osmoregulatory ability caused by lice, or a direct effect of lice on osmoregulation. The low II of chalimus lice may suggest the first option as the most likely explanation.

### Pre-adult lice elevated liver size and plasma osmolality in female trout

There was a physiological effect of salmon louse infection on female sea trout osmoregulation at a mean lice II of 0.16 lice g^−1^ when the louse had developed into the pre-adult stage, 28 days after infection. This II is equal to the pre-adult II reported to affect physiology in Atlantic salmon (Fjelldal *et al.,* 2020), but lower than the pre-adult II reported to affect sea trout (Wells *et al.,* 2006) and Arctic char (Fjelldal *et al.,* 2019), where 0.3 lice g^−1^ is the reported threshold for physiological consequences.

The present study is the first to show an effect of fish sex on the response to pre-adult lice. At the pre-adult stage, un-infected fish of both sexes had developed normal hypo-osmoregulatory ability. Increased HSI and plasma osmolality in female trout suggests that pre-adult lice impose more pronounced osmoregulatory and metabolic consequences in female compared to male trout. Salmon louse infection has been shown to elevate liver glycogen level in sea trout when the lice reach the pre-adult stage (Wells *et al.,* 2006). Since liver glycogen binds water (Mackay and Bergman, 1932), elevated liver glycogen could increase the HSI. The rapid decrease in HSI 48 h following the shift from seawater to freshwater in females further suggests that this is related to liver water content. Accordingly Wells *et al.* (2007) found that liver glycogen peaked in lice infected trout when the lice reached the pre-adult stage and decreased again following change from seawater to freshwater.

There were stronger responses in plasma glucose and lactate in male compared to female trout over the current simulated pre-mature return to freshwater. Hence, females may have been better adapted for the change from salt water to freshwater. If this is transferable to wild sea trout, that would suggest more female than male trout among louse infected trout that returns pre-maturely to freshwater. The current stronger physiological consequences of salmon louse in female compared to male trout further supports this notion.

### Pre-adult lice did not elevate plasma cortisol in female trout

Lice infection gave more pronounced and clear changes in plasma osmolality than plasma cortisol in the current study. There was, however, a significant interaction between treatment and sex on plasma cortisol, caused by high plasma cortisol in infected females and low plasma cortisol in infected males. Nonetheless, pre-adult lice gave elevated plasma osmolality but not cortisol in female trout. Bjørn *et al.* (2001) found a positive relationship between louse II and plasma cortisol in wild sea trout, where an II of >1.6 lice g^−1^ gave higher cortisol compared to < 0.7. Similarly, Bjørn and Finstad (1997) found elevated plasma cortisol when the lice reached the pre-adult stage in 2-year-old first-generation hatchery reared brown trout post-smolts infected with salmon louse with an II of 0.8 lice g^−1^. Wells *et al.* (2007) estimated that II >0.3 lice g^−1^ with pre-adult lice induced a response in plasma cortisol in wild caught anadromous brown trout that were infected with salmon louse in the laboratory.

The elevation in plasma cortisol works in concert with elevated plasma ions and osmolality (Bjørn and Finstad, 1997; Bjørn *et al.,* 2001; Wells *et al.,* 2007), and positive correlations between plasma osmolality and cortisol have been reported in salmon louse infected Atlantic salmon (Fjelldal *et al.,* 2020). In that study the authors suggested that the lice induced elevation of plasma cortisol may be a pure stress response and/or a protective mechanism to maintain ionic homeostasis, since cortisol can both elevate epithelial membrane permeability (Bonga, 1997) and increase gill NKA enzyme activity (McCormick *et al.,* 2008). The current low louse II affected hypo-osmoregulatory ability more severely than plasma cortisol, which may indicate that lice induced cutaneous lesions are the main cause for the elevated plasma ion levels in the host.

### Development of hypo-osmoregulatory ability in hatchery reared sea trout

Before migration to seawater, sea trout needs to smoltify—a collective term for the morphological, physiological and behavioral changes that pre-adapt the fish for movement from fresh- to saltwater (Parry, 1960; Hogstrand and Haux, 1985; Tanguy *et al.,* 1994; Lysfjord and Staurnes, 1998; Ugedal *et al.,* 1998; Nielsen *et al.,* 2006).

In the current experiment, 2-year-old hatchery-reared F1 offspring of wild sea trout had elevated plasma ion levels and osmolality after 72 h in 34 ppt seawater, but achieved full hypo-osmoregulatory ability after a prolonged period at 34 ppt. Wild migrating sea trout smolts also seems to adjust their hypo-osmoregulatory ability after entering seawater. Seawater challenge tests (33–34 ppt) on sea trout first time migrant smolts have shown mean plasma levels of 160 mM after 24 h (Lysfjord and Staurnes, 1998) and 134–146 mM after 72 h (Ugedal *et al.,* 1998). Indeed, Lyse *et al.* (1998) captured sea trout post-smolts in gillnets at a mean distance from shore at 4.3–8 m and mean depth of 0.6–0.8 m, which was explained by a preference to shallow water caused by osmoregulatory problems in high-salinity deep water.

Parry (1960) postulated that sea trout needs to be physiologically prepared by the smoltification process in order to survive in full-strength seawater, while Tanguy *et al.* (1994) concluded that smoltification in brown trout is not as well developed as in Atlantic salmon, and is not necessary for seawater adaption. Following the approach of Jonsson *et al.* (1994), water salinity was increased from 0.8 to 34 ppt over a 5-day period to support a gradually adjustment of the osmoregulatory machinery in the current study, and there were no mortalities during this period. Nonetheless, the presently studied fish had a mean plasma chloride level of 158 mM after the 5-day salinity adjustment and in addition 72 h at 34 ppt, which was associated with an increase in cumulative mortality to 3.3%. Ugedal *et al.* (1998) released two-year-old hatchery reared F1 offspring of wild anadromous brown trout 2.5 km upstream from the river mouth. Following the release, downstream migrating trout were captured in a fish trap and subjected to 72 h and 34 ppt seawater challenge tests, which gave plasma Cl^−^ values ranging between 135 and 147 mM. Only 34% of the released hatchery reared trout migrated downstream, and 72 h and 34 ppt seawater challenge test on the whole populations before release into the river gave mean plasma Cl^−^ values that varied from 164 to 167 between years. Total mortality in those tests was 1.7%. Fish shorter than 18 cm in body length were not able to regulate their plasma Cl^−^ below 160 mM, which was set as a threshold plasma Cl^−^ level to separate fish with and without an acceptable seawater tolerance. Similarly, in the present study, fish shorter than 18 cm had plasma Cl^−^ equal to or higher than 160 mM, but also several larger fish had plasma Cl^−^ above 160 mM as also observed by Ugedal *et al.* (1998).

### Slower adaption of hypo-osmoregulatory ability in male trout

In the current study, totally 54% of the sampled fish had plasma Cl^−^ below 160 mM after 72 h at 34 ppt; 60% of the females and 44% of the males. After 14 days at 34 ppt, plasma Cl^−^ levels were 130 mM in un-infected females, which were lower than in un-infected males (148 mM). The same trends were observed for plasma Na^+^ and osmolality, which suggests that females had established full hypo-osmoregulatory ability after 14 days, while males had not. Un-infected males needed a further 18 days to reach full hypo-osmoregulatory ability. Hence, a lice infection after 1 month in full strength seawater should be included at the currently used experimental settings. Such an approach would assess the physiological impact of salmon louse in trout with fully developed hypo-osmoregulatory ability, as in wild sea trout at river descendance. Indeed, Jonsson *et al.* (1994) retained hatchery reared brown trout for 0, 2, 4 and 8 weeks in seawater tanks before release into the natural seawater habitat and found increased recapture rate for those fish released after 4 and 8 weeks.

Both sexes showed a similar reduction in plasma ions when changed from salt water to freshwater (the simulated premature river ascendance) in the current study, but males showed an increase in plasma glucose and lactate. The observed sexual dimorphism in development of hypo-osmoregulatory ability in seawater and response in plasma glucose and lactate at change from seawater to freshwater may be related to growth rate and sexual maturation (Dellefors and Faremo, 1988; Jonsson and Jonsson, 1993). The observed higher CSI in male compared to female trout may further suggest a link to sexual maturation (Järvi *et al.,* 1991). In general, plasma osmolality was lower after return to freshwater compared to before the fish entered seawater. The reason behind this reduction is most likely the increase in freshwater temperature between the time points S1–S5 (Nakari, 1997).

## Conclusions

Female trout were better adapted for changes in water salinity, while male trout were more robust against infection with salmon louse. If this is transferable to wild sea trout populations, it may suggest a skewed sex ratio against more females among louse infected wild sea trout that returns pre-maturely to freshwater. However, possible effects of sexual maturation need to be studied in more detail in both sexes. The currently observed harmful effects of salmon louse on female trout may suggest that the elevated salmon louse infection pressure generated by salmon farming have strong and unexplored negative effects on reproduction in wild sea trout populations. Further research on this topic is highly relevant for the conservation of wild sea trout populations.

## Data availability

The data underlying this article are available in the article and in its online supplementary material (Supplementary file 5).

## Supplementary Material

Web_Material_coac078
