# Supplementary figures and images for: In sea trout, the physiological response to salmon louse is stronger in female than in males
Source: Conserv Physiol. 2023 Jan 12;11(1):coac078. doi: 10.1093/conphys/coac078 (PMC9835072; doi:10.1093/conphys/coac078)

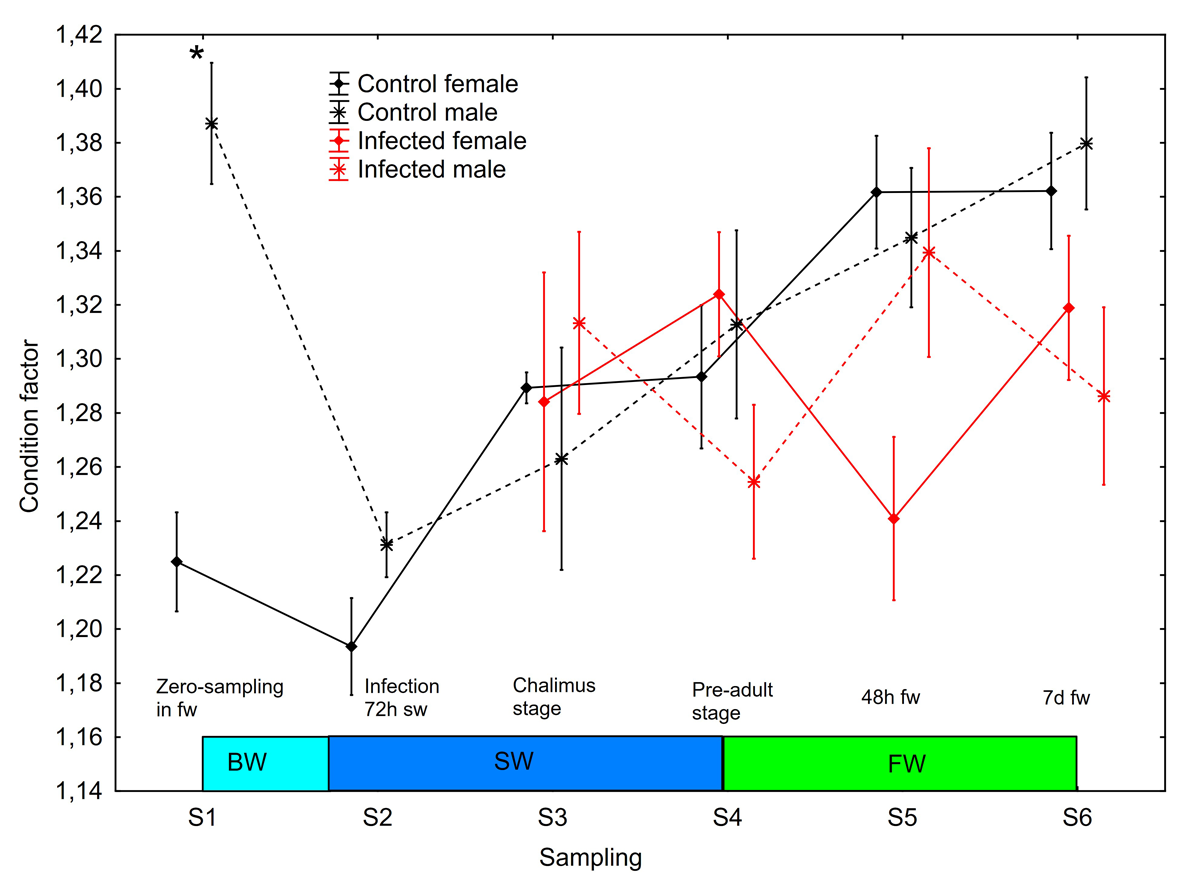

Supplement: Web_Material_coac078 [file web_material_coac078.zip › Resub Supplementary file 1_.tif]

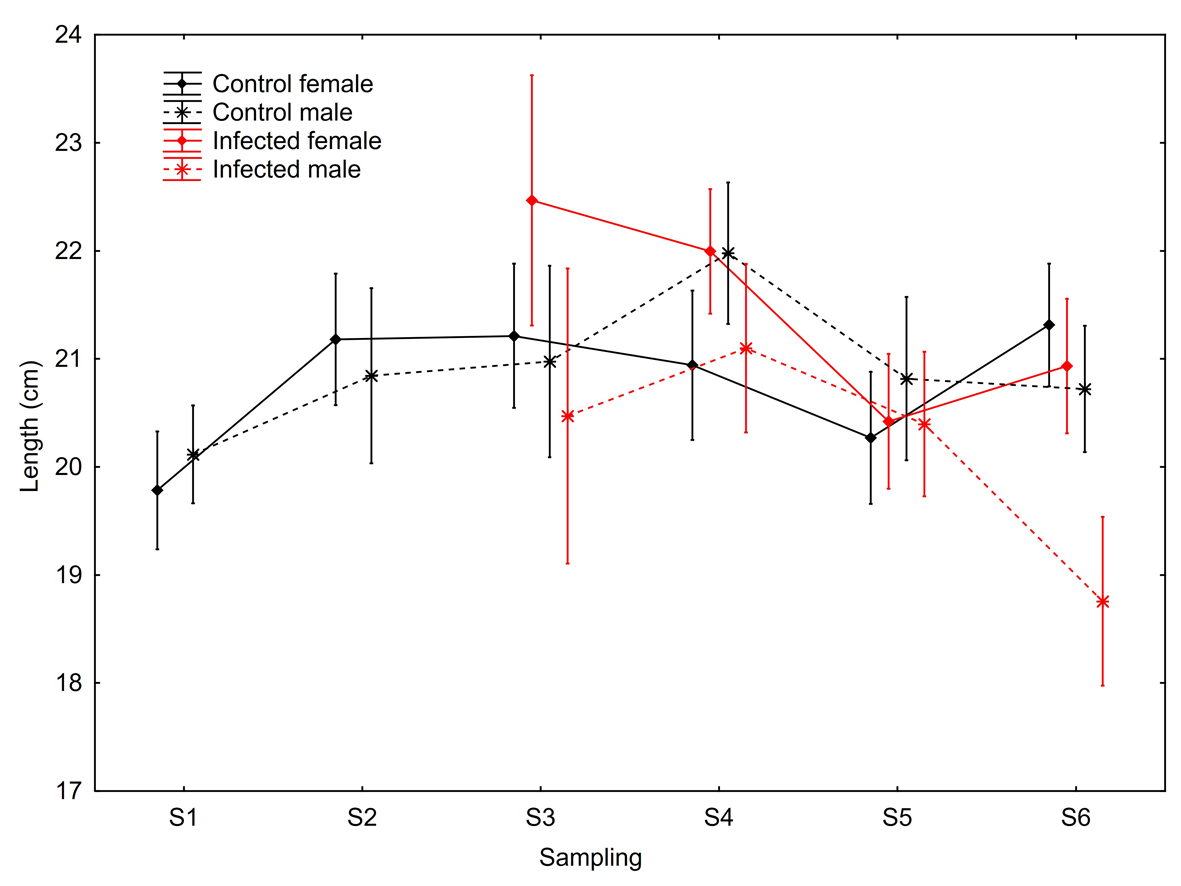

Supplement: Web_Material_coac078 [file web_material_coac078.zip › Resub Supplementary file 2_.tif]

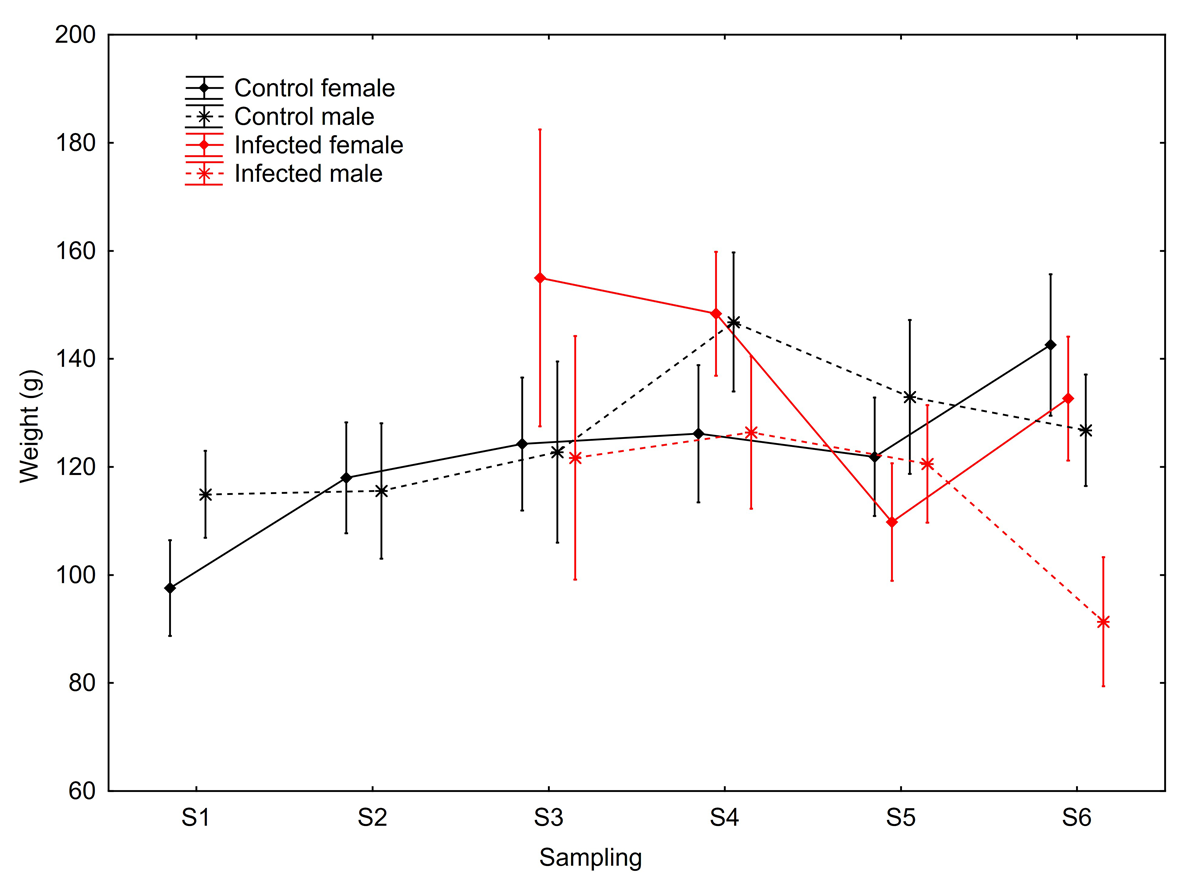

Supplement: Web_Material_coac078 [file web_material_coac078.zip › Resub Supplementary file 3_.tif]

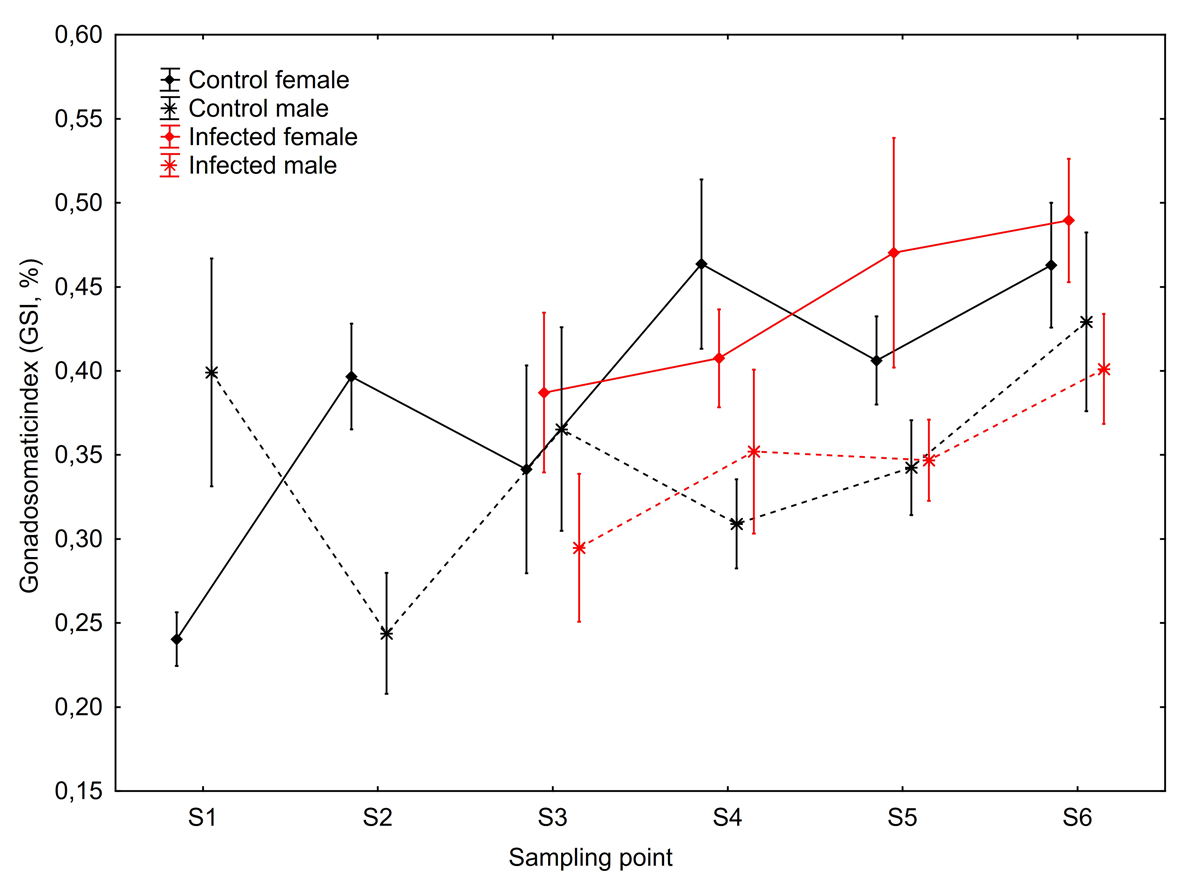

Supplement: Web_Material_coac078 [file web_material_coac078.zip › Resub Supplementary file 4_.tif]
